# Tear Film Interferometry, Meibography, and Optical Coherence Tomography Angiography for Rosacea

**DOI:** 10.3390/diseases14030105

**Published:** 2026-03-12

**Authors:** Matteo Capobianco, Marco Zeppieri, Federico Visalli, Francesco Pellegrini, Leandro Inferrera, Rosa Giglio, Irene Gattazzo, Francesco Cappellani, Fabiana D’Esposito, Caterina Gagliano

**Affiliations:** 1 Eye Clinic, Catania University, Policlinico G. Rodolico, 95121 Catania, Italy; capobiancoteo@gmail.com (M.C.);; 2 Department of Ophthalmology, University Hospital of Udine, 33100 Udine, Italy; 3 Department of Medicine, Surgery and Health Sciences, University of Trieste, 34127 Trieste, Italy; 4 Department of Ophthalmology, ASFO Pordenone Hospital, 33170 Pordenone, Italy; 5 Department of Ophthalmology, Ospedale Sant’Antonio, Azienda Ospedaliera, 35127 Padova, Italy; 6 Department of Medicine and Surgery, University of Enna “Kore”, Piazza dell’Università, 94100 Enna, Italy; 7 Mediterranean Foundation “G.B. Morgagni”, 95125 Catania, Italy; 8 Imperial College Ophthalmic Research Group (ICORG) Unit, Imperial College, London NW1 5QH, UK

**Keywords:** rosacea, ocular rosacea, meibography, lipid layer thickness, OCT angiography, tear film

## Abstract

Background/Objectives: Rosacea is a chronic inflammatory dermatosis that may involve the eye, causing surface and adnexal damage that can precede cutaneous signs. Detecting subclinical ocular changes is clinically important because early ocular surface dysfunction may be missed on routine examination yet progress to corneal complications, allowing earlier preventive management when identified. We prospectively evaluated subclinical ocular alterations in cutaneous rosacea using a combined, fully non-invasive high-tech imaging workflow—tear film interferometry, infrared meibography, and exploratory retinal optical coherence tomography angiography (OCT-A)—including patients without clinically evident ocular involvement. Methods: Sixteen patients with cutaneous rosacea (mean age 44.3 ± 11.2 years; 4 males, 12 females) were enrolled and divided into: Group 1—rosacea with clinically evident ocular involvement (*n* = 11); Group 2—rosacea without clinical ocular involvement (*n* = 5). Six age-matched healthy subjects served as controls (Group 3). All underwent LipiView II^®^ interferometry and meibography to quantify lipid-layer thickness (LLT, nm) and meibomian gland (MG) loss score (1 = normal–4 = severe), plus retinal OCT-A (Optovue Inc., Fremont, CA, USA). ANOVA with post hoc Tukey test assessed inter-group differences. Results: OCT-A showed no significant alterations in superficial or deep retinal plexuses across groups (*p* > 0.05). Conversely, LLT was significantly reduced in both rosacea groups vs. controls (OD: 45.5 ± 21.4 nm and 67.4 ± 10.1 nm vs. 92.7 ± 8.2 nm; OS: 40.4 ± 15.3 nm and 66.4 ± 10.1 nm vs. 96.0 ± 6.7 nm; *p* < 0.001). MG score was markedly higher (worse) in rosacea (OD: 3.63 ± 0.50 and 3.20 ± 0.83 vs. 1.83 ± 0.75; OS: 3.45 ± 0.68 and 3.40 ± 0.54 vs. 1.66 ± 0.81; *p* < 0.001). Ocular symptoms were reported by 85% of patients yet slit-lamp examination revealed surface alterations in 58% of asymptomatic cases. Conclusions: Tear film interferometry and meibography detect early ocular surface impairment in rosacea—even in the absence of clinical signs—while retinal microvasculature appears unaffected. Routine ophthalmologic screening of all rosacea patients could enable prompt treatment of subclinical dysfunction, potentially preventing corneal complications. Retinal OCTA metrics were not significantly different in this small pilot cohort, and these negative findings should be interpreted cautiously pending larger studies.

## 1. Introduction

Rosacea is a chronic, relapsing inflammatory disorder that primarily affects the skin, although ocular involvement is also well recognized [1,2,3]. Cutaneous manifestations typically involve the central face, beginning with episodic flushing and often progressing to persistent erythema, papules, pustules, telangiectasias, and, in some patients, phymatous changes [4,5]. Ocular disease is common and may range from conjunctival and eyelid abnormalities to corneal involvement in more severe cases [6,7]. Taken together, these features can have a substantial impact on quality of life [1,5]. The pathophysiology of rosacea is thought to reflect the interplay of neurovascular dysregulation, innate immune activation, Demodex overpopulation, and environmental triggers. Increased expression of pro-inflammatory peptides, enhanced kallikrein-related peptidase 5 activity, and the presence of Demodex folliculorum have all been implicated in sustaining inflammation, while UV exposure, heat, spicy foods, alcohol, and emotional stress are among the most commonly reported triggers of disease flares [8,9,10,11].

From an ophthalmic standpoint, the classical manifestations of ocular rosacea are well established, whereas the earlier, subclinical phase remains less clearly characterized, particularly at the level of the ocular surface and retinal microvasculature [6,12]. In this setting, objective imaging may offer a meaningful advantage. Tear film interferometry provides a non-invasive estimate of lipid-layer thickness (LLT), while infrared meibography allows direct visualization of meibomian gland (MG) structure and dropout [13,14]. Across both adult and pediatric studies, meibography has proven useful in identifying structural abnormalities, whereas interferometry-derived LLT has shown greater variability across clinical contexts [7,12]. For this reason, the assessment of meibomian gland dysfunction (MGD) is likely to be more informative when symptoms, lid-margin findings, and noncontact meibography are interpreted together rather than in isolation [13,14].

Against this background, multimodal non-invasive imaging—including tear film interferometry, infrared meibography, and optical coherence tomography angiography (OCT-A)—offers a practical approach to evaluating anterior-segment dysfunction and, more exploratorily, possible posterior-segment vascular changes [13,14,15,16]. OCT-A provides dye-free visualization of the retinal and choroidal microvasculature together with quantitative metrics and has increasingly been used to investigate vascular abnormalities across a range of ocular conditions [15,16]. Because ocular rosacea is frequently under-recognized [17,18,19,20], objective testing may help identify tear film and meibomian dysfunction even when slit-lamp findings are limited [7,12,18,21,22]. Within the same imaging workflow, OCT-A also makes it possible to explore the posterior segment [22,23,24]. However, studies combining tear film interferometry, infrared meibography, and retinal OCT-A remain limited, especially in patients without clinically evident ocular involvement; accordingly, OCT-A was included here as an exploratory endpoint, supported by previous OCTA observations in erythematotelangiectatic rosacea [23]. The aim of this prospective pilot study was to quantify LLT and MG morphology, compare these parameters among rosacea patients with and without clinically evident ocular involvement and age-matched healthy controls, and explore macular and optic-disc vessel density in the superficial and deep plexuses by OCT-A [17,18,19,20,23].

## 2. Materials and Methods

### 2.1. Study Design and Participants

We carried out a prospective observational study at the Eye Center “G.B. Morgagni-DSV” in Catania, Italy. We consecutively enrolled 16 adults with dermatologist-confirmed cutaneous rosacea (4 males/12 females; mean age 44.26 years) and 6 age-matched healthy controls. Adult patients with dermatologist-confirmed cutaneous rosacea were referred from the dermatology unit to our ophthalmology clinic and consecutively screened for inclusion. Given the pilot, feasibility-oriented nature of the study and the limited available data to support a robust a priori effect-size assumption for combined interferometry/meibography and OCT-A in cutaneous rosacea—particularly in patients without clinically evident ocular involvement—no formal sample size calculation was performed. Therefore, we enrolled a consecutive series of eligible rosacea patients and age-matched controls available during the study period. The primary purpose of this pilot cohort was to provide preliminary estimates of magnitude and variability for LLT, meibography MG loss, and OCT-A metrics to inform sample-size planning for adequately powered future studies; OCT-A analyses were considered exploratory. All examinations were non-invasive and part of a routine diagnostic work-up.

The study adhered to the principles of the 1964 Declaration of Helsinki and its later amendments. The protocol was reviewed by the Ethics Committee of the University Hospital of Catania, which confirmed that formal IRB approval was not required because of the purely observational design, the absence of experimental drugs, and the exclusive use of non-invasive procedures. Written informed consent was obtained from all participants prior to enrolment.

The dermatologic diagnosis, including subtype classification (erythematotelangiectatic or papulopustular), was established by a dermatologist according to the revised criteria of the National Rosacea Society Expert Committee. In this pilot cohort, only erythematotelangiectatic and/or papulopustular rosacea were represented; given the small sample size, no subtype-stratified analyses were prespecified. We included patients aged 18–65 years with Fitzpatrick skin phototypes I–IV who had been on a stable systemic therapy regimen for at least 3 months before enrolment. Fitzpatrick phototype was recorded at enrolment and is reported descriptively; no phototype-stratified analyses were prespecified, given the pilot sample size.

Exclusion criteria were: history of ocular surgery, contact lens wear, autoimmune or connective tissue disease, diabetes mellitus, pregnancy, and use of topical or systemic medications known to affect the tear film (such as isotretinoin, cyclosporine, or anticholinergics) within the previous three months. We also excluded eyes with retinal pathology that could interfere with OCT-A interpretation. No standardized tear-stability tests (Tear Film Break-Up Time/Non-Invasive Break-Up Time or TBUT/NIBUT) and no validated symptom questionnaires (e.g., Ocular Surface Disease Index or OSDI) were collected.

Sixteen consecutive patients meeting these criteria were stratified into three groups:

- Group 1 (*n* = 11): rosacea with manifest ocular involvement (blepharitis, conjunctival hyperemia, and/or punctate corneal defects);
- Group 2 (*n* = 5): rosacea without clinically evident ocular signs or symptoms;
- Group 3 (*n* = 6): six age-matched healthy volunteers without a history of ocular surface disease or systemic inflammatory disorders, who served as controls;

As this was a single-visit, cross-sectional study, we did not systematically collect data on the chronology of cutaneous versus ocular symptom onset; therefore, no inference can be drawn from our dataset about which manifestation preceded the other. Also, because this was a pilot study with consecutive recruitment, groups were not size-matched a priori; therefore, the final group sizes reflect the distribution of ocular findings in the referred rosacea population.

### 2.2. Ocular Surface Imaging

Tear film interferometry (LipiView II^®^, Johnson & Johnson Vision, Irvine, CA, USA) was used to quantify lipid-layer thickness (LLT, nm) and MG status via white-light interferometry to assess the interference pattern of the tear film lipid layer. Briefly, LipiView II^®^ uses white-light interferometry to provide a non-invasive quantitative estimate of the tear film lipid-layer thickness (LLT, reported in nm), which serves as an objective surrogate of the lipid component of the tear film. In addition, the system provides infrared meibography imaging to visualize MG architecture and dropout. Patients were instructed to blink naturally with each eye while a 20 s video sequence was recorded, and the mean LLT (nm) was automatically calculated by the proprietary software throughout the recording. Measurements exhibiting severe blinking artifacts or inadequate fixation were eliminated and redone. Infrared meibography of the upper and lower eyelids was conducted in a single session under consistent room illumination and without the application of topical anesthesia [14,25]. Following the method of Arita et al., gland loss was quantified by evaluating the central two-thirds of the tarsal plate, with partial glands (score 0–3) and gland dropout (score 0–2) analyzed separately. The MGD grading scales used (marginal telangiectasia, orifice plugging, lid margin irregularity and thickening, partial glands, gland dropout) were developed and validated with standardized images, with inter-observer reliability ranging from 0.36 to 0.87 and intra-observer reliability from 0.49 to 0.93 (kappa) [25]. Two masked evaluators individually assessed meibography pictures; in instances of discord, a consensus grade was established following a collaborative assessment. All LipiView II^®^ acquisitions were performed by two trained operators following the same standardized protocol.

### 2.3. Retinal OCT-Angiography

The AngioVue^®^ OCT-A system (RTVue XR Avanti, Optovue Inc., Fremont, CA, USA) was utilized to evaluate retinal microvasculature. OCT-A is a dye-free OCT-based technique that generates en face maps of the perfused retinal microvasculature by detecting motion contrast from moving blood cells. In this study, we used OCT-A to estimate vessel density (%) as a quantitative measure of perfused vasculature within the scanned area in the superficial and deep capillary plexuses.

Following pupil dilatation with topical tropicamide 1% as required, 3 × 3 mm scans of the macula and optic disc were obtained, centered on the fovea and disc, respectively. Only scans with an automated quality index (QI) ≥ 7/10 and minimal motion artifacts were included in the analysis. Automated segmentation was employed to define the superficial capillary plexus (extending from the internal limiting membrane to the inner plexiform layer) and the deep capillary plexus (ranging from the inner nuclear layer to the outer plexiform layer). Vessel density (%) was extracted using the AngioAnalytics^®^ software (version: 2018.1.1.63), for both plexuses across the whole 3 × 3 mm scanning area. All images were reviewed by a retina specialist to identify segmentation errors; scans exhibiting substantial artifacts or erroneous segmentation were excluded and, whenever feasible, required. All OCT-A acquisitions were performed by two trained operators using a standardized protocol. Images were reviewed by a retina specialist for segmentation or motion artifacts; scans with substantial errors were excluded and re-acquired when feasible.

### 2.4. Slit-Lamp Examination

A masked ophthalmologist performed a comprehensive slit-lamp examination of the eyelids, conjunctiva, cornea, and tear meniscus height to exclude coexisting ocular surface or anterior segment disease. Lid margin findings were graded according to the criteria proposed by Arita et al.: telangiectasia crossing the meibomian orifices (0–3), orifice plugging (0–3), margin irregularity (0–2), and margin thickening (0–2), with operational definitions based on the number and distribution of abnormalities and the presence of “notching” or a rounded contour [25].

### 2.5. Statistical Analysis

Data are expressed as mean ± SD. All tests were two-sided. Given the pilot nature and the unbalanced group sizes, no multivariable adjustment for potential confounders (e.g., age or sex) was performed; OCT-A analyses were considered exploratory. Inter-group differences were tested using a one-way ANOVA with Tukey post hoc. When the overall ANOVA was significant, Tukey’s post hoc test was used for pairwise comparisons among groups. The significance level was set at α = 0.05. *p* < 0.05 was considered significant (SPSS v26.0). No a priori sample size calculation was performed due to the exploratory, pilot design. No formal test–retest repeatability/reproducibility analysis was planned or performed in this pilot dataset; therefore, because of the absence of repeated measurements, intraclass correlation coefficients (ICC) were not computed.

## 3. Results

Fitzpatrick phototypes I–IV were represented in the study population. Given the pilot sample size, we did not perform phototype-stratified comparisons of ocular surface or OCT-A parameters.

### 3.1. Lipid-Layer Thickness and Meibography

**Figure 1:**
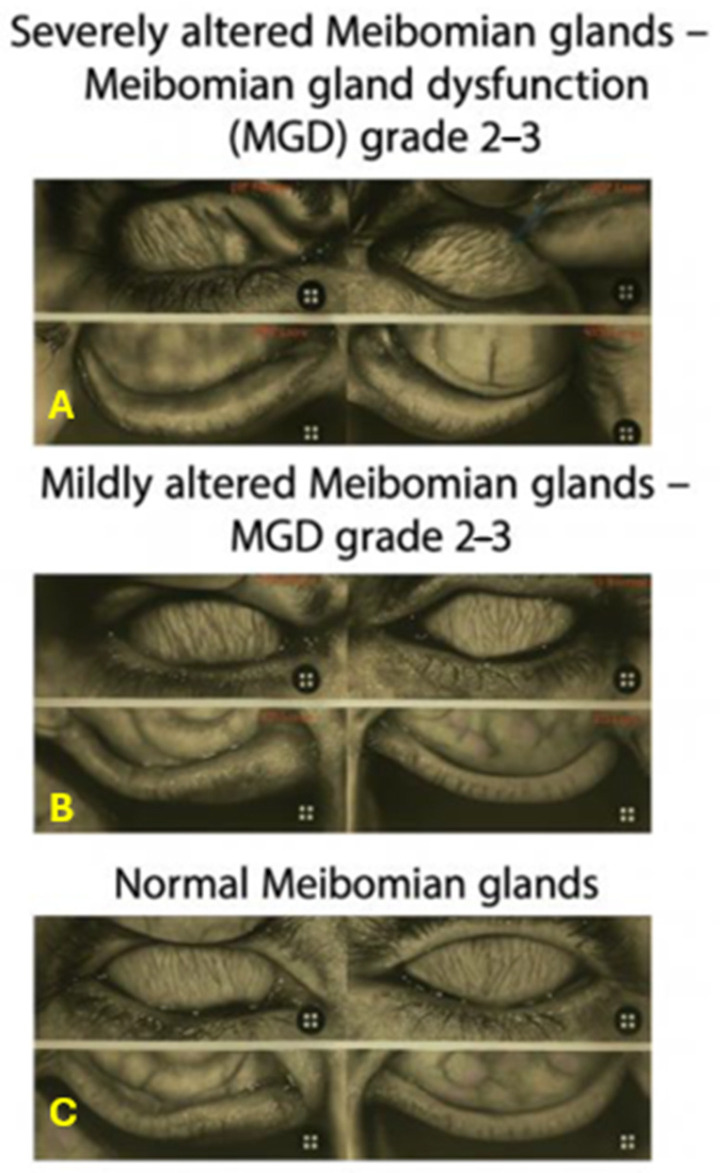
Infrared meibography images among research groups. Representative infrared meibography of the upper and lower eyelids. (**A**) Severely altered meibomian glands—meibomian gland dysfunction (MGD) grade 3–4—typical of rosacea with overt ocular involvement. Severe meibomian gland loss characterized by truncation and distortion, indicative of ocular rosacea (**B**) Mildly altered meibomian glands—MGD grade 2–3—observed in rosacea patients without clinical ocular signs. Mild to severe gland shortening and partial dropout noted in rosacea absent ocular symptoms. (**C**) Normal meibomian glands (grade 0–1) from healthy controls. Normal parallel gland architecture in healthy individuals.

Table 1 summarizes LLT and MG scores. One-way ANOVA showed significant overall differences among the three groups for both LLT and MG scores in both eyes (all *p* < 0.001). Tukey post hoc analysis showed that LLT was markedly lower in Group 1 compared to Group 2 in OS (*p* = 0.03), whereas OD exhibited a comparable but non-significant trend (*p* = 0.08). In contrast, MG scores did not significantly differ between the two rosacea subgroups. Representative meibography images of severe, mild, and normal glands are shown in Figure 1.

The MG dropout scores were severely increased accordingly [7,26]. No difference emerged between Group 1 and Group 2. In adults with rosacea, meibography shows significant MG loss and worse MGD grading compared with healthy controls [7]; mechanistically, ocular surface inflammation can impair MG structure and function [26]. Representative meibography images of severe, mild, and normal glands are shown in Figure 1.

### 3.2. Retinal Vasculature

While rosacea is not traditionally linked to retinal vascular disease, OCT-angiography has been utilized to investigate the potential extension of systemic neurovascular inflammation beyond the ocular surface. No significant differences were detected in superficial or deep vessel density among the three groups (*p* > 0.05). Nevertheless, prior OCTA studies in erythemato-telangiectatic rosacea reported increased flow in the outer retinal layer and choriocapillaris despite unchanged vessel densities in the superficial and deep capillary plexuses [23]. Overall, these findings indicate that posterior segment microvascular alterations may be present even in asymptomatic ocular conditions. The lack of retinal changes in this group supports the notion that rosacea-associated vascular dysregulation predominantly affects superficial tissues.

### 3.3. Clinical Findings

Ocular symptoms (dryness, burning, photophobia) were reported by 85% of patients, yet slit-lamp alterations of the corneoconjunctival surface were present in 58% of otherwise asymptomatic cases. This is consistent with reports indicating that ocular involvement is common in rosacea (≈58–72%) and that the temporal relationship between ocular and cutaneous manifestations is heterogeneous; ocular signs have been reported to precede cutaneous manifestations in a minority of patients (up to 20%) [6].

## 4. Discussion and Future Direction

Our results confirm that tear film LLT is significantly reduced and MG dropout is markedly increased in rosacea patients—even in the absence of overt ocular inflammation.

A reduction in LLT similar to that observed in our cohort has already been reported in ocular rosacea by Blackie et al. using interferometry, and was associated with higher non-invasive breakup time values and worse Ocular Surface Disease Index scores [13]. Histologic and in vivo confocal studies further show that, in rosacea, MG acinar epithelial cells are affected by perilobular lymphocytic infiltration, keratinisation, and atrophy [26,27]. In this context, the prevalence of grade 3–4 alterations on meibography in our series closely reflects these underlying structural changes and strengthens the view that MGD and evaporative dry eye are among the earliest, often subclinical, ocular manifestations of cutaneous rosacea [6,7]. This is clinically relevant, because early MG impairment may sustain ocular surface inflammation and, if left unrecognized, may eventually contribute to corneal involvement [5,26].

By contrast, OCT-A did not show significant differences in macular or peripapillary vessel density between rosacea and control eyes. Although these results must be interpreted in light of the relatively small cross-sectional sample, they suggest that in uncomplicated cutaneous rosacea with mild-to-moderate ocular involvement, the disease burden may remain largely limited to the ocular surface and periocular vasculature, rather than reflecting a broader posterior-segment microangiopathy. This differs from conditions such as psoriasis, lupus erythematosus, and atopic dermatitis, in which OCT-A may reveal parafoveal capillary rarefaction [28,29]. Current evidence suggests that rosacea-related vasculopathy is primarily driven by neurovascular dysregulation affecting the superficial dermal plexus and periocular tissues; in this setting, dermal endothelial cells may overexpress vascular endothelial growth factor, substance P, and calcitonin gene-related peptide, thereby promoting persistent vasodilatation without necessarily involving the deeper ocular vascular beds [10,11]. From this perspective, the absence of OCT-A abnormalities in our cohort is more consistent with predominantly superficial or periocular vascular involvement than with a diffuse pan-ocular microangiopathy. At the same time, a recent OCT-A study in erythematotelangiectatic rosacea described subtle retinal microvascular changes, suggesting that posterior-segment involvement may still be present in a subset of patients [23]. Overall, the available evidence does not currently support intensified posterior-segment surveillance in all otherwise low-risk rosacea patients, although larger longitudinal OCT-A studies are still needed before more definitive conclusions can be drawn.

These findings also carry clear practical implications. Interventions such as thermal pulsation devices, intense pulsed-light (IPL) therapy, or low-dose oral doxycycline have been shown to improve the quality of MG secretion and reduce ocular symptoms in rosacea [30,31,32]. Our data suggest that LLT < 70 nm or a meibography grade ≥ 2 may function as effective benchmarks for detecting early lipid layer deficit—even in asymptomatic patients—potentially averting sight-threatening keratitis. The combination of reduced LLT and increased MG dropout in patients with mild-to-moderate cutaneous rosacea—including those without significant ocular complaints—is in line with previous studies linking rosacea to MGD, structural gland loss, and evaporative tear film dysfunction [7,33,34]. Taken together, this supports a proactive ophthalmic approach in patients with cutaneous rosacea, with particular value placed on non-invasive interferometry and infrared meibography to identify subclinical MGD and tear film instability before more severe corneal complications emerge [7,12,33,34,35,36]. Quantitative LLT measurements and MG dropout scores, both of which correlate with other signs of MGD and may improve after targeted treatment [36,37,38], may also serve as useful objective biomarkers for monitoring response to lid hygiene, warm compresses, vectored thermal pulsation, IPL, and the systemic or topical anti-inflammatory and antimicrobial therapies commonly used in ocular rosacea [39,40]. In routine practice, serial imaging could therefore help tailor treatment intensity and duration to the patient’s structural and functional course, rather than relying only on symptoms, which are known to correlate imperfectly with objective dry-eye findings [35,36,37,38,39,40,41].

Lastly, the clinical findings are coherent with the imaging data. In our cohort, ocular symptoms were common but slit-lamp signs involving the corneoconjunctival surface were also documented in patients who were otherwise asymptomatic. This reinforces an important point: ocular rosacea may easily be under-recognized when evaluation is based only on symptoms or on routine slit-lamp examination. For this reason, our findings support the use of a structured ocular surface assessment even in patients who do not report overt ocular complaints [6].

### 4.1. Strengths and Limitations

Strengths of this study include its prospective design, the use of objective multi-modal imaging (tear film interferometry, meibography and OCT-A) and the inclusion of both symptomatic and asymptomatic rosacea patients. Conventional tear function assessments, including Schirmer and tear breakup time, were excluded from this study due to its emphasis on objective imaging biomarkers; nonetheless, their incorporation in future research may yield supplementary functional data. Limitations include a small sample size, the lack of a validated symptom questionnaire, and the absence of tear cytokine or matrix metalloproteinase analysis, which could have clarified inflammatory pathways [42]. In particular, the rosacea-without-clinically-evident-ocular-involvement subgroup was small (*n* = 5), and group sizes were unbalanced, with an uneven sex distribution across groups. The sample size also did not allow a meaningful analysis of meibomian parameters by cutaneous rosacea subtype. A further limitation is that a standardized cutaneous severity score (e.g., RASI) was not collected, preventing stratified analyses by rosacea severity. These features may introduce sampling bias and limit external validity, and the dataset does not allow meaningful adjustment for potential confounders. Accordingly, the OCT-A analysis should be considered exploratory and potentially underpowered in this pilot cohort. Moreover, given the cross-sectional design and small sample size, we may have been underpowered for OCTA endpoints; in addition, OCTA metrics can vary with age and sex, and the limited sample did not allow adjustment for these factors. Longitudinal studies with larger cohorts—especially before and after systemic therapies such as doxycycline or isotretinoin—are warranted to determine whether improvement in cutaneous disease parallels restoration of LLT and MG morphology and to explore the potential role of antimicrobial/anti-inflammatory agents on ocular surface homeostasis. As a limitation, we did not conduct a specific test–retest repeatability experiment (intraclass correlation coefficient), which may be particularly relevant given the small sample size.

### 4.2. Future Directions

Subsequent research should corroborate these findings in larger multicenter cohorts with extended follow-up and a wider range of illness severity. Longitudinal investigations using tear film interferometry, meibography, OCT-A, and in vivo confocal microscopy could elucidate the temporal interactions among ocular surface inflammation, meibomian gland remodeling, and cutaneous disease activity. A key objective is to ascertain if early targeted interventions—such as intense pulsed light therapy, thermal pulsation, or low-dose doxycycline—can reinstate lipid layer thickness, mitigate gland dropout, and enhance long-term ocular results. Integrating imaging biomarkers with tear film inflammatory markers may facilitate the identification of specific ocular rosacea endotypes and promote earlier collaborative management between dermatologists and ophthalmologists.

**Figure 2:**
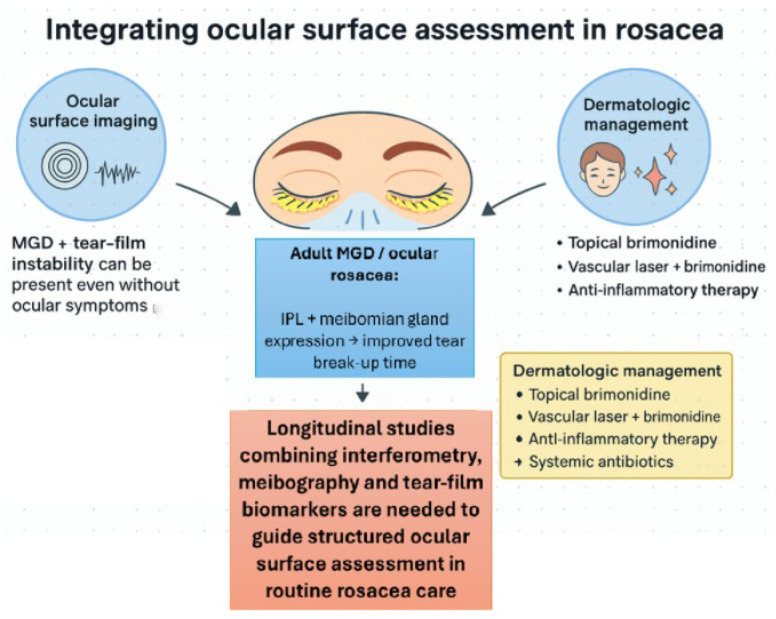
Integrating ocular surface assessment in rosacea. High-resolution interferometry and infrared meibography can reveal subclinical MGD and tear film instability in patients with rosacea, even in the absence of ocular symptoms. In adults with ocular rosacea, these findings may prompt targeted interventions such as intense pulsed light (IPL) combined with (MG expression to improve tear break-up time. The figure also highlights the need for longitudinal studies combining interferometry, meibography, and tear film biomarkers to guide structured ocular surface assessment in routine rosacea care, alongside dermatologic management with topical brimonidine, vascular laser plus brimonidine, anti-inflammatory therapy, and, when indicated, systemic antibiotics.

Figure 2 illustrates how ocular surface imaging, targeted treatment of MGD, and dermatologic therapy can be combined into an integrated management strategy for rosacea.

In this pilot cohort, tear film interferometry and infrared meibography consistently detected ocular surface involvement in patients with cutaneous rosacea, including those with no clinically evident ocular disease. Compared with healthy controls, lipid layer thickness was reduced and MG dropout was increased, whereas OCT-A metrics did not show significant differences across groups. In line with previous reports, our data support a simple point that matters in everyday practice. MGD and tear film instability can appear early and may be overlooked if we rely only on symptoms or slit-lamp findings [7,12]. This is not just a subclinical curiosity—MGD is a key mechanism of evaporative dry eye in ocular rosacea, and recognizing it before corneal involvement develops creates a practical opportunity for earlier intervention [1,4]. On this basis, our results argue for a more systematic role of non-invasive interferometry and meibography in the routine assessment of rosacea patients, as objective tools that can guide monitoring and management.

This study was not designed to evaluate treatment efficacy; however, identifying subclinical MGD helps frame a mechanism-oriented approach when intervention is appropriate. Available evidence suggests that IPL combined with MG expression can improve tear film parameters and, in some settings, symptom scores in MGD populations that include ocular rosacea [30]. Pediatric series further indicate that structured regimens integrating lid hygiene, warm compresses, topical therapies, and systemic antibiotics may improve gland morphology and tear stability while limiting corneal sequelae [32]. More broadly, rosacea care remains multidisciplinary: dermatologic management targets inflammation and neurovascular dysregulation, which are central to the disease process [43,44], and vascular-directed strategies for persistent erythema or telangiectasias—such as topical brimonidine, with laser-based approaches in selected cases—can reduce cutaneous disease burden [45]. Looking ahead, longitudinal studies combining imaging with tear film inflammatory biomarkers may clarify whether improvement in cutaneous disease parallels restoration of lipid-layer metrics and may help refine pragmatic thresholds for initiating ocular treatment [46]. Until such data are available, incorporating a structured ocular surface assessment into rosacea care appears justified to detect early dysfunction, support proactive management, and reduce the risk of vision-threatening complications [1,4]. Larger, adequately powered longitudinal studies are also needed to confirm our findings and to determine whether subtle posterior-segment OCT-A changes emerge in specific rosacea subgroups.

More broadly, evidence from cutaneous rosacea shows that high-resolution non-invasive imaging can reveal early or subtle disease features through clinicodermoscopic correlations, reinforcing the rationale for applying objective imaging modalities (interferometry and meibography, with exploratory OCT-A) to detect subclinical ocular involvement in pilot cohorts [47].

## 5. Conclusions

The clinical interpretation of our findings offers a practical message for routine care. Subclinical MGD and tear film instability can develop early in rosacea; however, they can be easily overlooked and/or missed when assessment relies solely on symptoms and slit-lamp examination. In this context, the use of tear film interferometry along with infrared meibography can provide both quantitative and structural information that can enhance the standard assessment and facilitate the detection of ocular surface involvement even in the absence of clinically evident ocular pathology. In contrast, exploratory OCT-A analysis showed no significant differences in retinal vascular density in this pilot cohort. Under the conditions of the present study, this suggests that the posterior segment is unlikely to be the primary site of rosacea-related ocular involvement.

This imaging profile, from a clinical management perspective, supports a more proactive approach: patients with cutaneous rosacea may benefit from ocular surface screening even in the absence of obvious ocular symptoms. The observed reduction in objective lipid layer thickness, combined with meibographic evidence of glandular loss, is consistent with an evaporative dry eye pathway and suggests the need for early preventive intervention focused on the lid margin and MG function, before corneal complications emerge. Overall, these findings also reinforce the importance of an interdisciplinary approach to rosacea. Close coordination between dermatologists and ophthalmologists remains essential for early recognition of ocular surface involvement and intensifying treatment when indicated.

## Figures and Tables

**Table 1:** Lipid-layer thickness (LLT) and meibomian gland (MG) atrophy scores among study cohorts. Interferometric LLT and MG dropout scores in rosacea patients with ocular involvement, rosacea patients without ocular involvement, and age-matched healthy controls. Values are mean ± SD; OD = right eye, OS = left eye; *p* values derived from one-way ANOVA.

Parameter	Rosacea + Ocular (*n* = 11)	Rosacea Ocular-Free (*n* = 5)	Controls (*n* = 6)	*p* ANOVA
LLT OD (nm)	45.5 ± 21.4	67.4 ± 10.1	92.7 ± 8.2	<0.001
LLT OS (nm)	40.4 ± 15.3	66.4 ± 10.1	96.0 ± 6.7	<0.001
MG score OD	3.63 ± 0.50	3.20 ± 0.83	1.83 ± 0.75	<0.001
MG score OS	3.45 ± 0.68	3.40 ± 0.54	1.66 ± 0.81	<0.001

Values are expressed as mean ± standard deviation (SD. *p* values are obtained from one-way ANOVA with Tukey post hoc analyses. Group 1 compared to Group 3: *p* < 0.001; Comparison between Group 2 and Group 3: *p* < 0.001; Group 1 versus Group 2: not significant;

## Data Availability

Raw data were generated at the Ophthalmology Department, San Marco Hospital, Medical University, Catania, Italy. Data available on request due to restrictions (e.g., privacy, legal or ethical reasons).
